# Supplementary material for: Enhanced Antioxidant Effects of the Anti-Inflammatory Compound Probucol When Released from Mesoporous Silica Particles
Source: Pharmaceutics. 2022 Feb 24;14(3):502. doi: 10.3390/pharmaceutics14030502 (PMC8953917; doi:10.3390/pharmaceutics14030502)
Supplement: Supplementary file 1 [file pharmaceutics-14-00502-s001.zip › pharmaceutics-1599238-supplementary.pdf]

pharmaceutics

Supplementary Materials: Enhanced Antioxidant Effects of the Anti-inflammatory Compound Probucol when Released from Mesoporous Silica Particles

Michael Lau, Benjamin Sealy, Valery Combes, Marco Morsch and Alfonso E. Garcia-Bennett

Table S1. Structural and textural data of AMS-6 particles utilized in this work. The unit cell parameter, a_o, was obtained from powder X-ray diffraction. The average pore volume, average pore size and surface area (P_{vol}, $P_{size,}$ and $SA_{BET,}$ respectively) were obtained from nitrogen sorption measurements. Reduction in the pore size and surface area of NH_2-AMS-6 compared to CAL-AMS-6 was due to the presence of the functional propylamine groups within the internal surface of NH_2-AMS-6 MSP. Further reduction in pore volume and surface area was observed in the FITC-AMS-6 when compared to NH_2-AMS-6 indicating the presence of FITC within the mesopores. The HD_{size} was measured in PBS buffer, whilst the ζ-potential was measured in distilled water. The ζ-potential of CAL-AMS-6 was negative due to the presence of surface silanol groups. The NH_2-AMS-6 MSP showed the highest hydrodynamic (HD_{size}) particle size due to particle agglomeration, and a positive ζ-potential due to the presence of amine groups.

MSP	a_o, Å	$P_{vol,}$ cm³/g	P_{size}, Å	SA_{BET} m²/g	HD_{size}, nm (± STD)	ζ-potential, mv (± STD)
AS-AMS-6	115.3	-	-	-	594 (8)	36.3(3.0)
CAL-AMS-6	114.8	0.75	46.7	777.3	564(18)	28.5(0.6)
NH_2-AMS-6	118.8	0.39	37.9	470.5	749(24.1)	29.6(0.8)
FITC-AMS-6	100.7	0.35	36.2	401.5	782 (25.1)	30.8(0.4)

Pharmaceutics **2022**, *14*, 502. https://doi.org/10.3390/pharmaceutics14030502 www.mdpi.com/journal/pharmaceutics

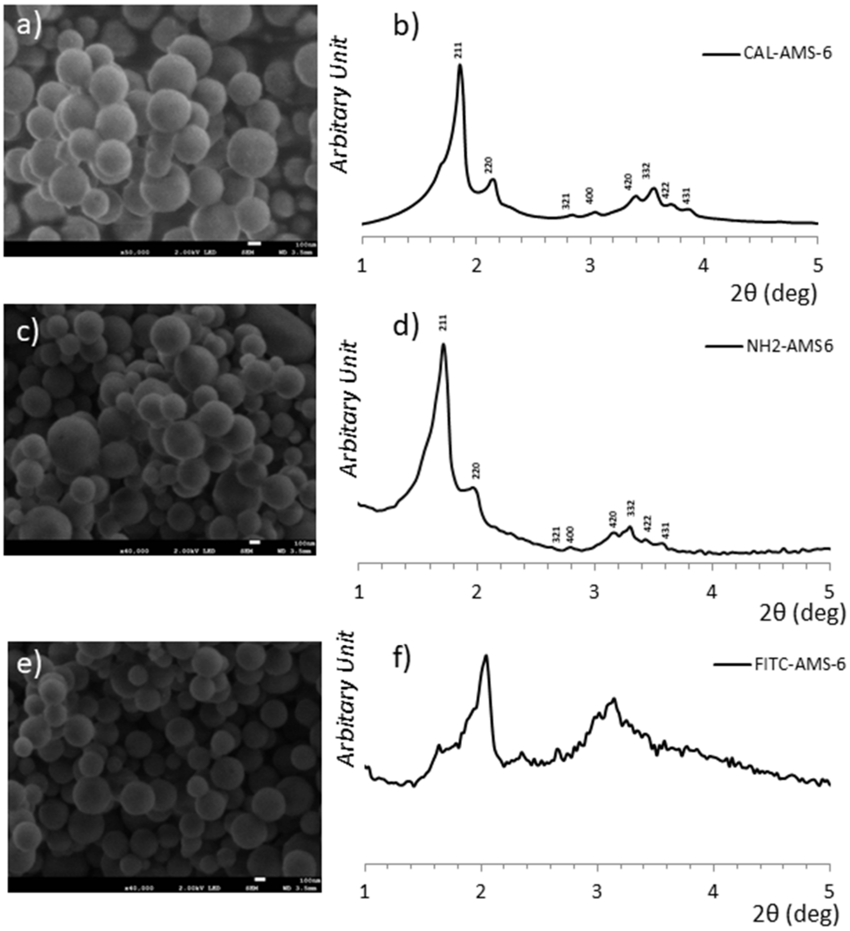

Figure S1. SEM and XRD scans of mesoporous materials, (**a**,**b**) CAL-AMS-6, (**c**,**d**) NH_2-AMS-6, (**e**,**f**) FITC-AMS-6.

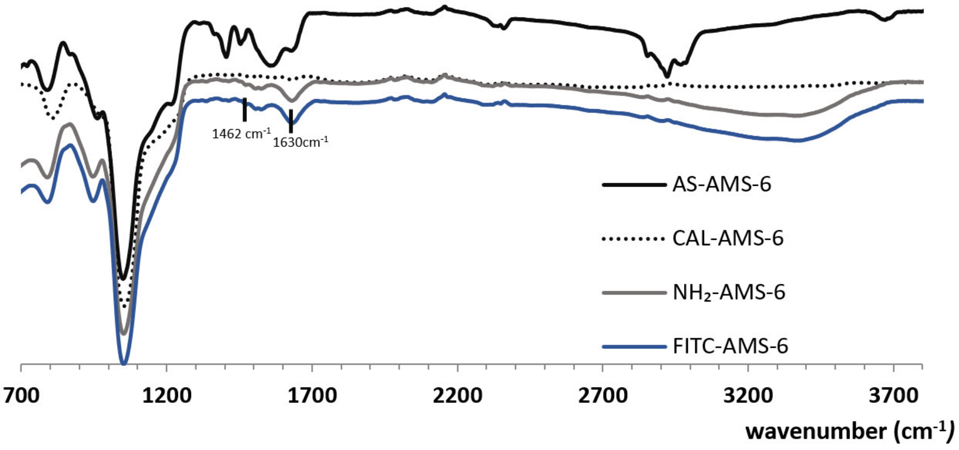

Figure S2. FT-IR spectra of the as-synthesised MSP (AS-AMS-6), CAL-AMS-6, NH_2-AMS-6, and FITC-AMS-6 materials. The absorption bands of the 1000–1200 cm^{-1} (Si-O-Si asymmetric stretching), 965 cm^{-1} (Si-O stretching) and 801 cm^{-1} (Si-O-Si symmetric stretching) is present in all MSP and represent the peaks of the silica framework [1]. The absorption peak at 1462 cm^{-1} is attributed to the reaction between the amino groups (-NH_2) on NH_2-AMS-6 and the isothiocyanate groups (-N=C=S) from FITC [2]. The presence of NH_2 groups in NH_2-AMS-6 and FITC-AMS-6 materials is attributed

to the stretching band at 1630 cm^{-1} [2]. The broad band between 3200 and 3700 cm^{-1} is attributed to the O-H bond stretching of the surface silanol groups and the adsorbed water molecule [3].

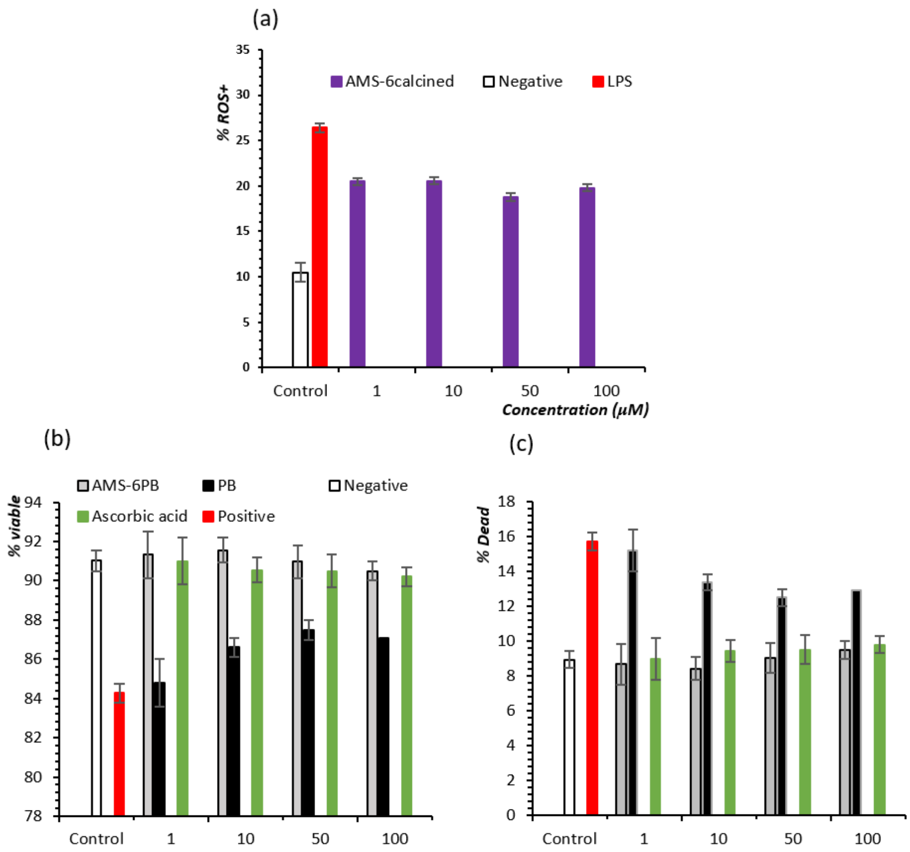

Figure S3. (**a**) Concentration dependent effects of mesoporous silica AMS-6 on the generation of ROS+ HBEC cells after 24 h incubation with 1 μg/mL LPS. (**b**,**c**) Percentage of viable and dead cells after addition of PB, AMS-6PB or ascorbic acid after 24 h incubation with 1 μg/mL LPS. The positive control (red bar) are cells incubated with 1 μg/mL LPS and negative control (white bar) is cells incubated with media only for 24 h.

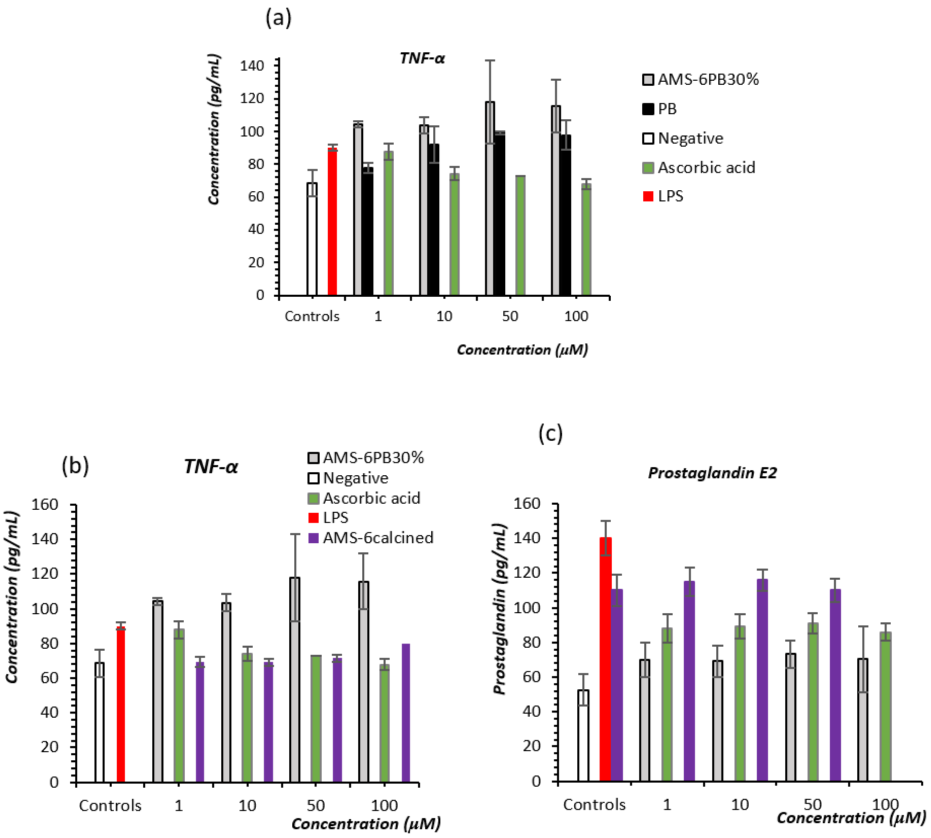

Figure S4. (**a**) Concentration dependent effects of the test compounds on the generation of TNF-α in HBEC cells after 24 h incubation with 1 µg/mL LPS. (**b**) Concentration dependent effects of mesoporous silica AMS-6, AMS-6PB and ascorbic acid on the generation of TNF-α in HBEC cells after 24 h incubation with 1 µg/ml LPS. (**c**) Concentration dependent effects of mesoporous silica AMS-6, AMS-6PB and ascorbic acid on the generation of prostaglandin E_2 in HBEC cells after 24 hours incubation with 1 µg/mL LPS. The positive control (red bar) are cells incubated with 1 µg/mL LPS and negative control (white bar) is cells incubated with media only for 24 h.

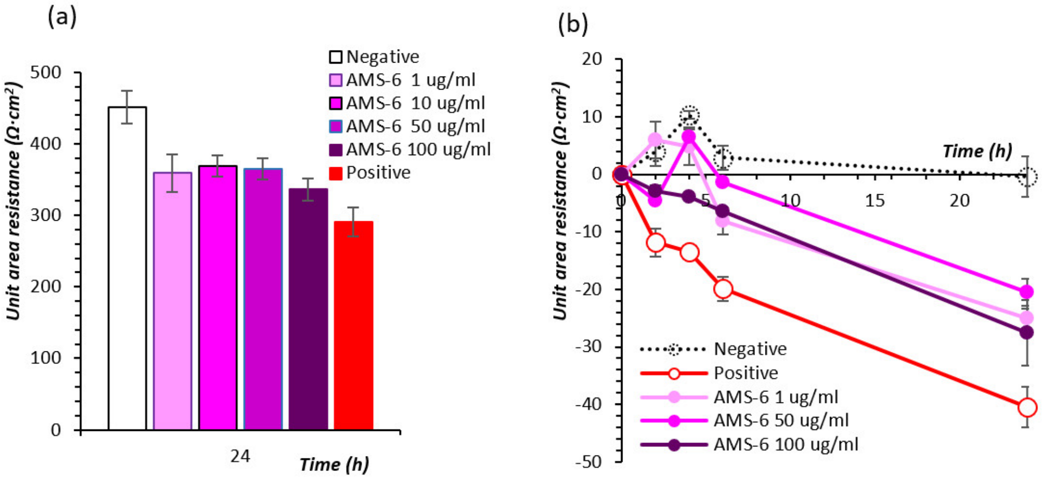

Figure S5. (**a**) Absolute TEER electrical resistance measurements conducted on monolayers of HBEC as a BBB integrity model incubated with 1 µg/mL LPS and mesoporous silica AMS-6 particles alone after 24-h incubation. (**b**) Time dependent normalized resistance measurements at 1 µM, 50 µM and 100 µM mesoporous silica particles of AMS-6 alone with 1 µg/mL LPS, compared to controls.

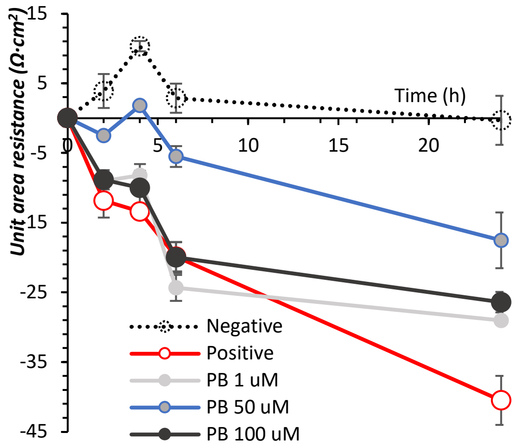

Figure S6. Time dependent normalized resistance measurements at 1μM, 50μM and 100μM for PB, with 1 μg/mL LPS, compared to controls.

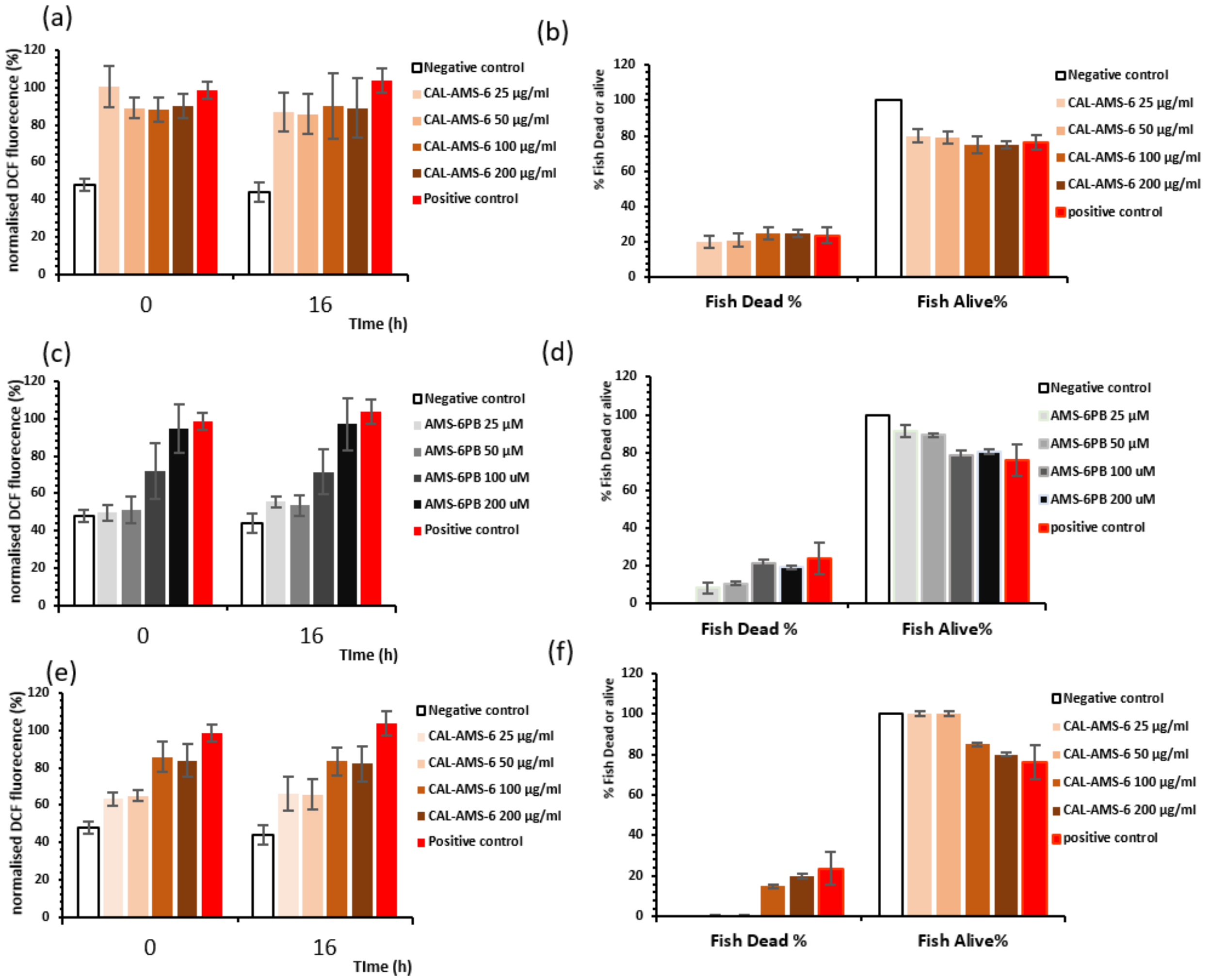

Figure S7. (**a**) Measured DCF fluorescence intensity in zebrafish embryo (2dpf) pre-treated with CAL-AMS-6 at different concentrations for 24 h, 5 mM H_2O_2 for 1 h, and 25 μM DCFDA for 45 min. (**b**) Percentage of fish dead or alive (*n* =12) at the end of the DCF measurement (16 h) as observed under bright field microscopy. (**c**) Measured DCF fluorescence intensity in zebrafish following pre-treatment with test compound AMS-6PB at different concentrations for 24 h, 5 mM H_2O_2 for 1 h, and 25 μM DCFDA for 45 min. (**d**) Percentage of fish dead or alive (*n* =12) at the end of the DCF measurement (16 h) as observed under bright field microscopy. (**e**) Measured DCF fluorescence in zebrafish embryo pre-treated with CAL-AMS-6 at different concentrations, followed by 45 min

incubation with 25 μM DCFDA for 45 min. (**f**) Percentage of fish dead or alive (n =12) at the end of the DCF measurement (16 h) as observed under bright field microscopy. DCF measurement were normalised to the positive control and expressed as a percentage.

References

1. Simon, B.C.; Haudenschild, C.C.; Cohen, R.A. Preservation of endothelium-dependent relaxation in atherosclerotic rabbit aorta by probucol. *J. Cardiovasc. Pharmacol.* **1993**, *21*, 893–901.
2. Zheng, H.; Gao, C.; Che, S. Amino and quaternary ammonium group functionalized mesoporous silica: An efficient ion-exchange method to remove anionic surfactant from AMS. *Microporous Mesoporous Mater.* **2008**, *116*, 299–307.
3. Inoue, N.; Ohara, Y.; Fukai, T.; Harrison, D.G.; Nishida, K.i. Probucol improves endothelial-dependent relaxation and decreases vascular superoxide production in cholesterol-fed rabbits. *Am. J. Med. Sci.* **1998**, *315*, 242–247.
